# Adsorption of Cationic Pollutants from Water by Cotton Rope Coated with Cyclodextrin Polymers

**DOI:** 10.3390/polym14122312

**Published:** 2022-06-07

**Authors:** Ekkachai Martwong, Nathapong Sukhawipat, Jatupol Junthip

**Affiliations:** 1 Division of Science (Chemistry), Faculty of Science and Technology, Rajamangala University of Technology Suvarnabhumi, Phra Nakhon Si Ayutthaya 13000, Thailand; ekkachai.m@rmutsb.ac.th; 2 Division of Polymer Engineering Technology, Department of Mechanical Engineering Technology, College of Industrial Technology, King Mongkut’s University of Technology North Bangkok, Bangkok 10800, Thailand; nathapong.s@cit.kmutnb.ac.th; 3 Faculty of Science and Technology, Nakhon Ratchasima Rajabhat University, Nakhon Ratchasima 30000, Thailand

**Keywords:** adsorption, cyclodextrin, citric acid, poly (vinyl alcohol), cotton cord, methylene blue, paraquat, crystal violet, Langmuir isotherm

## Abstract

The contamination from perilous organic compounds (pesticide and dyes) in water generates a significant problem for the environment and humans. A modified textile was prepared by a coating of anionic cyclodextrin polymer, obtained from the cross-linking between citric acid and β-cyclodextrin in the presence of poly (vinyl alcohol), on the cotton cord for cationic pollutant removal from an aqueous solution. Its physicochemical properties were also characterized by gravimetry, titration, stereomicroscopy, SEM, TGA, ^13^C NMR, and ATR-FTIR. The CC2 system exhibited 79.2% coating yield, 1.12 mmol/g COOH groups, 91.3% paraquat (PQ) removal, 97.0% methylene blue (MB) removal, and 98.3% crystal violet (CV) removal for 25 mg/L of initial concentration. The kinetics was fitted to the pseudo-second-order model using 6 h of contact time. The isotherm was suitable for the Langmuir isotherm with a maximum adsorption of 26.9 mg/g (PQ), 23.7 mg/g (MB), and 30.3 mg/g (CV). After 120 h of contact time in water and 5% *v*/*v* of HCI in ethanol, the weight loss was 7.5% and 5.6%, respectively. Finally, the recyclability performance reached 84.8% (PQ), 95.2% (MB), and 96.9% (CV) after five reuses.

## 1. Introduction

Clean water and sanitation are part of the sustainable development goal 6 (SDG 6), which is an essential point in socioeconomic development and will enhance water quality by reducing the contamination of water [1,2]. The presence of organic cationic contaminants including dyes or pesticides pollutes water, which affects the ecosystems and humans. The adsorption process is an ecofriendly technology to solve water pollution and various innovative adsorbents have been elaborated from sustainable and cost-effective resources [3]. This method has gained significant attention in recent years because this process provides many advantages such as ease of operation, minimal investment costs, reusability of adsorbent, low energy requirement, selectivity of adsorbent, and applicability for technology transfer [4]. The disadvantage of this method is related to the cost of regeneration (solvent use or energy consumption), the elimination of the adsorbent after its end of life, or the cost of reactants.

Paraquat (PQ) is a water-soluble agrochemical substance used to increase crop yield and safeguard the plants from pests. Nevertheless, this pesticide could menace both health [5,6,7,8] and the environment [9,10]. A concentration of paraquat of 0.1 mg/L has been the maximum permissible concentration for drinking water [11]. Different adsorbents have been elaborated for PQ adsorption such as kaolin [12], activated carbon [13], bentonite [14], cellulose nanofiber [15], biobased material [16,17,18], cyclodextrin polymers coated on textile [19,20], and cyclodextrin nanosponges [21,22,23,24]. Methylene blue (MB) is a hydrosoluble cationic dye with the chemical structure of thiazine that is used as a dye for the textile coloration, a medication for the treatment of methemoglobinemia, a stain for chromoendoscopy or different staining procedures, an antidote to potassium cyanide poisoning, a redox indicator in analytical chemistry, and a treatment for fungal infections in aquaculture [25]. The contamination of methylene blue in wastewater comes from textile, leather, cosmetics, photography, and other industries which could impact the environment and humans, because methylene blue is chemically stable, difficult to degrade, toxic, and carcinogenic according to its complex aromatic structures [26,27]. A concentration of methylene blue less than 1 mg/L has obviously been revealed as an aesthetic issue [28]. Various adsorbents have been prepared for MB removal such as hybrid composite of cyclodextrin/activated carbon [26], cyclodextrin/silver nanoparticles [29], hybrid adsorbent of cyclodextrin/silica [30], magnetic nanoparticles [31,32], cyclodextrin modified filter paper [33], cyclodextrin grafting wood flour [34], and insoluble cyclodextrin [35,36]. Crystal violet (CV) is a water-soluble cationic dye with the chemical structure of triarylmethane that is used as a dye, a histological stain, a topical antiseptic, and a dermatological agent [37]. Moreover, CV shows antifungal, antibacterial, antitumor, and other properties. The accumulation of CV in wastewater from various industries (textile, paper, plastics, printing, leather, paint, pharmaceutical, cosmetic, food, and paper industries) could affect humans [38] and the surroundings [39], because crystal violet is chemically firm, difficult to decompose, and toxic. A concentration of CV of 0.001 mg/L could be toxic and mutagenic to humans and animals [40]. Multitudinous adsorbents have been established for CV removal such as modified rice husk [41,42], tropical wild fern [43], polyamide nanofiber [44], magnetic nanoparticles [45,46], EDTA/graphene oxide functionalized corncob [47], EDTA/β-cyclodextrin insoluble [48], and cyclodextrin nanofiber [49].

Consequently, cyclodextrin-based adsorbents have been sparsely employed for environmental applications [50,51,52,53,54,55,56,57,58,59] because of the specific properties of cyclodextrin (CD) molecules which could entrap organic compounds with a suitable size into the cyclodextrin cavity to form an inclusion complex through host–guest interaction. Furthermore, citric acid (CTR) is an ecofriendly trifunctional cross-linking agent, which was reticulated with CD to obtain a three-dimensional cross-linked polymer for the enhancement of the adsorption performance of organic compounds [22,23,26,32,35,36]. Cellulose is a linear water-insoluble polysaccharide containing many glucose units, which is the most abundant renewable biopolymer on earth existing in the form of cotton, wood, and other fibers. According to its molecular structure, cellulose possesses good chemical reactivity, thermal behaviors, and mechanical properties [60]. Cellulose has also been reacted with CD and CTR via in situ polymerization to elaborate an effective adsorbent for aniline [61,62] and methylene blue removal [33,34]. Poly (vinyl alcohol) or PVOH, which has unique properties (high degree of swelling, biodegradability, and nontoxicity), has also been esterified with CD and CTR to build nanosponges for paraquat adsorption [22]. The presence of PVOH segments as new active sites could enhance the adsorption capacity according to the hydrogen bonding between PVOH and pollutants [63].

Nevertheless, the coating of β−CD with CTR in the presence of PVOH has never been applied on cellulose material and a β−CD/CTR/PVOH-functionalized cotton cord has never been reported for the adsorption of these three soluble cationic pollutants (PQ, MB, and CV). The objective of this work was to create an effective adsorbent for cationic contaminant removal. In this study, the coating of anionic cyclodextrin polymer, issued from the reticulation between CTR and β−CD in the presence of poly (vinyl alcohol), on a cotton rope was first investigated. Then, the physicochemical properties of the modified cord were also characterized by different techniques. Finally, an adsorption study with different parameters (pH of a solution, initial concentration of pollutants (PQ, MB, and CV) and time), a reusability study, and a stability study were examined.

## 2. Materials and Methods

### 2.1. Materials

β-cyclodextrin (Acros Organics, Geel, Belgium), poly (vinyl alcohol) M_w_ = 89,000–98,000 with 99+% hydrolyzed (Sigma-Aldrich, Saint Louis, MO, USA), citric acid monohydrate (RCI labscan, Bangkok, Thailand), cotton cord (Taisonghuad, Bangkok, Thailand), sodium hypophosphite (Acros Organics, Geel, Belgium), crystal violet (PanReac, Barcelona, Spain), methylene blue (CARLO ERBA Reagents S.A.S., Val de Reuil, France), and paraquat dichloride hydrate (Sigma-Aldrich, Saint Louis, MO, USA) were obtained from commercial sources. Ultrapure water was used for all experiments and other chemicals were analytical grade.

### 2.2. Preparation of Adsorbents

A 1 cm thick and 15 cm length of cotton rope was cleaned with hot water for 30 min, dried at 100 °C in a hot-air oven (UF10, Memmert), and measured as the initial mass (noted m_i_). After that, it was immersed into 100 mL of a mixture containing 10% *w*/*v* β−CD, 3% *w*/*v* sodium hypophosphite, the different quantities of CTR (2.5, 5 or, 10% *w*/*v*, and the different compositions of PVOH (0.1, 0.5, 1, or 2% *w*/*v*) under stirring (150 rpm) for 24 h at 30 °C, as noted in Table 1 for each formulation. The sample was drained before being placed on aluminum foil, heated at 140 °C for 90 min in a hot-air oven, and rinsed with hot water for 30 min to eliminate undesired products before drying at 100 °C in a hot-air oven. Ultimately, the modified cotton rope was weighed as the final mass (noted m_f_). The coating performance was expressed as a weight gain and calculated according to this equation:

(1) $$\mathrm{Weight}\;\mathrm{gain}\;\left(\%\right)=\frac{m_{f}-m_{i}}{m_{i}}\times 100\;$$

where m_i_ and m_f_ relate, respectively, to the cotton cord weight before and after the curing. Experiments were carried out in triplicate.

### 2.3. Characterization of Adsorbents

The physicochemical characteristic of the modified cotton cord was characterized by numerous methods. Fourier transform infrared spectroscopy (FTIR) experiments using attenuated total reflection (ATR) mode were performed on a Tensor 27 FTIR (Bruker, Billerica, MA, USA), which was accumulated from 64 scans in the 700–4000 cm^−1^ range with a resolution of 4 cm^−1^. The morphology of adsorbents was observed by a SMZ745T stereomicroscope (Nikon, Melville, NY, USA) linked with a DS-Fi3 digital camera. The thermogravimetric analysis (TGA) test was manipulated in an alumina pan with a Thermal Analyzer—STA 449 F3 (NETZSCH, Waldkraiburg, Germany) with a heating rate of 10 °C min^−^^1^ under nitrogen. ^13^C NMR (nuclear magnetic resonance) spectra were operated on an Ascend 400 WB spectrometer (Bruker, Billerica, MA, USA) at 100.62 MHz and 298 K using the magic angle spinning (MAS) technique, a delay time of 8 s, and a contact time of 1.5 ms. The scanning electron microscopy (SEM) observation was performed on a JEOL 6010 electron microscope (Tokyo, Japan) with an acceleration voltage of 15 kV.

The measurement of the ion exchange capacity (IEC) of adsorbents was executed by pH-metric titration. The modified cotton cord (0.1 g) was soaked into 50 mL of a 2% *w*/*v* calcium acetate solution for 24 h under agitation at 150 rpm. After sample elimination, the existing acetic acid solution was titrated by NaOH solution (0.05 M) using phenolphthalein as an indicator. The IEC was calculated in millimoles of COOH groups per gram of cotton cord using the following equation:

(2) $$\mathrm{IEC}\;(\mathrm{mmol}/g)=\frac{C_{\mathrm{NaOH}\;}\times {\;V}_{\mathrm{NaOH}}}{m}\;$$

where V_NaOH_ and C_NaOH_ correspond, respectively, to the equivalent volume (mL) and concentration (mol/L) of NaOH. The symbol m refers to cord weight (g). Experiments were run in triplicate.

The determination of the point of zero charge (PZC) of the modified cord was performed by pH-metric titration using the salt addition method. A 0.1 M NaCl solution with different pH from 3 to 10 using 0.1 M HCl and 0.1 M NaOH was first prepared. After that, 30 mL of each solution was filled into a bottle containing 50 mg of modified cord and it was agitated for 48 h at 150 rpm. The final pH of each solution was measured before calculating the ΔpH (the difference between the initial and final pH values). These ΔpH values were plotted versus the initial pH and the PZC was quantified at ΔpH = 0.

### 2.4. Adsorption Study

#### 2.4.1. Preliminary Adsorption Study

An amount of 10 mL of pollutant solution (PQ, CV, and MB) with a 25 mg/L of initial concentration at various pH (2, 3, 4, 5, 6.5, 8, 9, and 10), which was previously changed with 0.1 M HCl and 0.1 M NaOH, was filled to a test tube containing 50 mg of modified cord under stirring (150 rpm) for 360 min at 30 °C. The quantity of pollutants was measured by a GENESYS 10S UV–vis spectrophotometer (Thermo Scientific, Vantaa, Finland) at 257 nm, 590 nm, or 664 nm for PQ, CV, or MB respectively. The pollutant removal was calculated in percentage using the following equation:

(3) $$\%\;\mathrm{Removal}=\frac{{(C}_{0}-{\;C}_{t})}{C_{0}}\;\times \;100$$

where C_0_ and C_t_ relate, respectively, to the initial and real-time concentration of the contaminant. Experiments were executed in triplicate. The adsorption capacity (Q) was also expressed using the following equation:

(4) $$\mathrm{Adsorption}\;\mathrm{capacity}\;(\mathrm{mg}/g)=\frac{{(C}_{0}-C_{t})\;\times \;V}{m}$$

where C_0_ and C_t_ relate, respectively, to the initial and real-time concentration of the contaminant, V refers to the solution volume, and m stands for the cord mass.

#### 2.4.2. Kinetics Study

An amount of 10 mL of pollutant solution (PQ, CV, and MB) with a 25 mg/L initial concentration and optimal pH was added into a test tube containing 50 mg of modified cord under agitation of 150 rpm at various times (30, 60, 120, 180, 360 and 540 min) at 30 °C. The quantification of contaminants was described in the previous section. Experimental data were then fitted with two kinetics models:

Pseudo-first-order model:

ln (Q_e_ − Q_t_) = ln Q_e_ − k_1_t (5)

Pseudo-second-order model:

(6) $$\frac{t}{Q_{t}}=\frac{1}{k_{2}Q_{e}^{2}}+\frac{1}{Q_{e}}t$$

where Q_e_ and Q_t_ are the amount of pollutant adsorbed (in mg/g) at equilibrium and at time t, respectively, k_1_ (/min) and k_2_ (g/mg·min) are the adsorption rate constants, and t is the contact time (min). Experiments were investigated in triplicate.

The Chi-square test was used as a statistical analysis so as to evaluate the reasonableness of the kinetic models to the experimental data. The Chi-square value (χ^2^) was expressed by the following equation:

Chi-square value:

(7) $$\chi^{2}=\sum \frac{{{(Q}_{e,\exp}-{\;Q}_{e,\mathrm{cal}})}^{2}}{Q_{e,\mathrm{cal}}\;}$$

where Q_e,exp_ is the amount of pollutant adsorbed (in mg/g) at equilibrium calculated from the experimental data and Q_e,cal_ is the amount of pollutant adsorbed (in mg/g) at equilibrium estimated from the models.

The quantity of pollutant adsorbed against the square root of time was plotted using the intraparticle diffusion model as the following equation:

Intraparticle diffusion model:

Q_t_ = k_3i_t^0.5^ (8)

where Q_t_ is the quantity of the pollutant adsorbed (in mg/g) at time t, k_3i_ (g/mg·min^0.5^) is the adsorption rate constant, and t is the contact time (min). Experiments were performed in triplicate.

#### 2.4.3. Isotherm Study

An amount of 10 mL of pollutant solution with various initial concentrations (PQ (25, 50, 150, 250, and 300 mg/L), MB (25, 50, 150, 200, and 500 mg/L), and CV (25, 50, 150, 200, and 500 mg/L)) and optimal pH was filled into a test tube containing 50 mg of modified cord under agitation of 150 rpm at equilibrium and 30 °C. The measurement of the pollutants was previously described. Experimental data were then fitted with two isotherm models:

Langmuir isotherm:

(9) $$\frac{C_{e}}{Q_{e}}=\frac{1}{K_{L}Q_{m}}+\frac{C_{e}}{Q_{m}}$$

Freundlich isotherm:

(10) $${\ln\;\mathrm{Qe}=\ln\;K}_{F}+\frac{1}{n}\ln\;\mathrm{Ce}$$

where C_e_ is the equilibrium concentration of pollutant, Q_e_ is the amount of pollutant adsorbed (in mg/g) at equilibrium, Q_m_ is the theoretical maximum adsorption capacity (in mg/g), K_L_ is the Langmuir isotherm constant, K_F_ is the Freundlich isotherm constant, and 1/n is a heterogeneity factor.

The Chi-square test (Equation (7)) was also applied to the experimental data to assess the suitability of isotherm models.

#### 2.4.4. Stability Study

The stability of the treated cord was performed in various solvents such as water and in 5% *v*/*v* of HCI in ethanol. The functionalized cord (50 mg) was put into 10 mL of each solvent under agitation (150 rpm and 30 °C). At the desired time, this cord was evacuated, dried at 120 °C for 30 min, and finally weighed. The percentage of weight loss was expressed using the following equation:

(11) $$\%\;\mathrm{Weight}\;\mathrm{loss}=\frac{m_{i}-m_{d}}{m_{i}}\times \;100\;$$

where m_i_ and m_d_ relate, respectively, to the modified cord weight and decomposed cord weight. Experiments were performed in triplicate. Then, this textile was put back in the new solvent with the previous step before remeasuring the weight loss.

#### 2.4.5. Reusability Study

An amount of 10 mL of pollutant solution with 25 mg/L of initial concentration and optimal pH was added into a test tube containing 50 mg of treated cord under agitation of 150 rpm at equilibrium and 30 °C. The quantification of the pollutant was noted in the previous part. The adsorbent was then removed and regenerated by washing in 5% *v*/*v* of HCI in ethanol for pollutant desorption. After 6 h of immersion, the adsorbent was cleaned with ultrapure water for 30 min and reconditioned for the adsorption process.

## 3. Results and Discussion

### 3.1. Preparation and Characterization of Adsorbents

#### 3.1.1. Physicochemical Properties of Adsorbents

**Figure 1:**
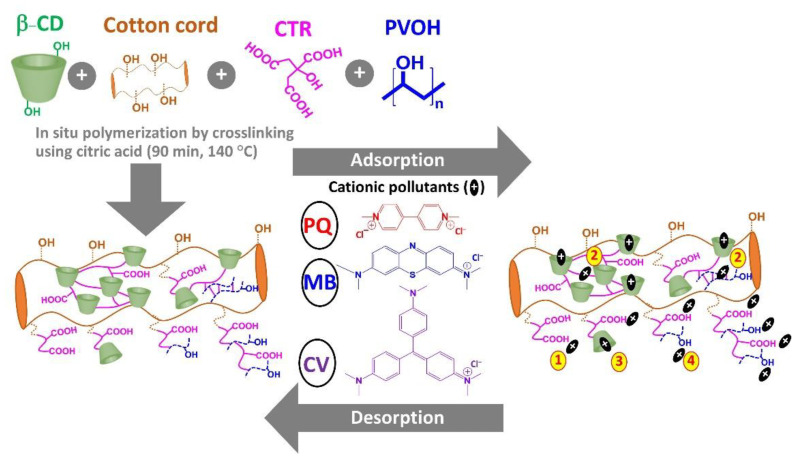
Coating of cyclodextrin polymers on the cotton rope and the probable adsorption ways with cationic pollutants.

The coating of anionic cyclodextrin polymer was efficaciously established on the surface of the cotton rope by in situ polymerization via an esterification reaction between hydroxyl groups (β−CD, PVOH, and/or cellulose) and carboxylic groups of CTR, as seen in Figure 1. The possible cross-linking reaction provided various forms such as CTR reticulated with cellulose, CTR reticulated with β−CD, CTR reticulated with PVOH, and CTR reticulated with PVOH, β−CD, and/or cellulose. The presence of available carboxylic groups could be dissociated to carboxylate groups which displayed the anionic character of functional textile and provided the adsorption towards cationic pollutants via electrostatic interaction (route i). It was possible to entrap cationic molecules in the cross-linked structure (route ii). The encapsulation of cationic pollutants also happened inside the β−CD cavity by host–guest interaction (route iii). Moreover, the appearance of PVOH segments offered the hydrogen bonding between hydrogen atoms of PVOH and nitrogen atoms of cationic species (route iv). So, the benefit of the PVOH addition proposed an attractive adsorption site for both the PVOH-reticulated structure and hydrogen-bonding resources on PVOH polymeric skeletons.

**Figure 2:**
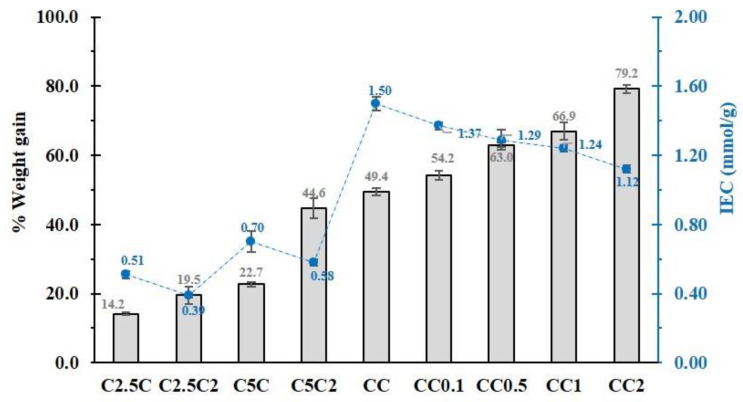
Weight gain and the ion exchange capacity of the modified cotton rope.

As seen in Figure 2, different coating formulations were investigated for the effect of the cross-linking agent of CTR and the PVOH addition on the physicochemical properties of modified cotton. For the modified cotton rope named C2.5C, C5C, and CC, the coating performance enhanced from 14.2% to 49.4% with an amount of CTR from 2.5% *w*/*v* to 10% *w*/*v*, which also expanded the ion exchange capacity from 0.51 mmol/g to 1.50 mmol/g because the superior quantity of CTR could improve the polyaddition between CTR and β−CD and/or cellulose, leading to a high coating rate and a prominent charge on the cotton surface. The addition of 2% *w*/*v* PVOH on a similar system, noted C2.5C2, C5C2, and CC2, displayed the same results, with the coating yield advancing from 19.5% to 79.2% and the ion exchange capacity from 0.39 mmol/g to 1.12 mmol/g. Nevertheless, the appearance of PVOH for each couple demonstrated a weight increase and a charge decrease because the cellulose from the cotton cord and β−CD had reacted previously with CTR while PVOH was esterified with available COOH groups from CTR to build the PVOH cross-linked structure and the PVOH connected segments, which gave a dominant coating efficiency although the loss of free COOH groups dropped the ion exchange capacity.

**Figure 3:**
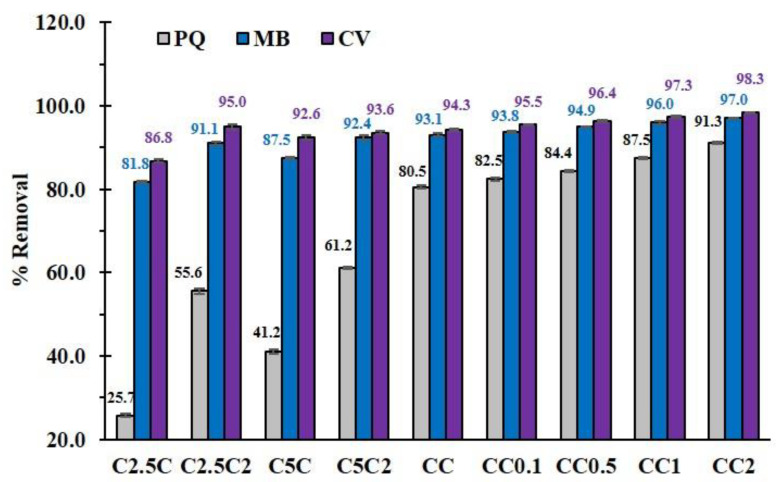
Adsorption of cationic pollutants (PQ, MB, and CV) on the modified cotton rope (conditions: 5 g/L of adsorbent dosage, 25 mg/L of pollutant initial concentration, optimal pH, and temperature at 303 K).

As observed in Figure 3, the adsorption of cationic pollutants (PQ (from 25.7% to 80.5%), MB (from 81.8% to 93.1%), and CV (from 86.8% to 94.3%)) raised with the amount of CTR from 2.5% *w*/*v* to 10% *w*/*v* according to the higher anionic charge on the surface to interact with cationic species. Furthermore, the removal rate of these cationic contaminants (PQ (from 55.6% to 91.3%), MB (from 91.1% to 97.0%), and CV (from 95.0% to 98.3%)) was also enhanced with the presence of PVOH for the concentration of CTR from 2.5% *w*/*v* to 10% *w*/*v*, respectively, due to the supplementary adsorption from hydrogen bonding. Consequently, the 10% *w*/*v* of CTR with the presence of PVOH in the formulation was a benefit for the coating performance and the adsorption with cationic pollutants. 

Herein, the different quantity of PVOH on 10% *w*/*v* of CTR in the formulation was investigated, as observed in Figure 2. The increase of PVOH from 0.1 to 2% *w*/*v* enlarged the coating rate from 49.4% to 79.2%, because the esterification of CTR with cellulose from a cotton cord was favorable as the solid support for the cross-linking between CTR and β−CD, and/or PVOH to create various structures. These results were in agreement with the literature in which a higher quantity of PVOH has shown a great opportunity, in cross-linking with pulp fibers, to enhance the mechanical properties [64]. However, the addition of PVOH from 0.1 to 2% *w*/*v* dropped the ion exchange capacity from 1.50 mmol/g to 1.12 mmol/g because of the extinction of free COOH functions from CTR which was attached to PVOH segments. This circumstance resulted in the improvement of adsorption towards cationic pollutants (PQ (from 82.5% to 91.3%), MB (from 93.8% to 97.0%), and CV (from 95.5% to 98.3%)) with the increase of PVOH from 0.1 to 2% *w*/*v*, as seen in Figure 3. This result has been reported in the literature with the increase of PVOH in cyclodextrin polymers improving the efficiency of aniline extraction [65].

**Figure 4:**
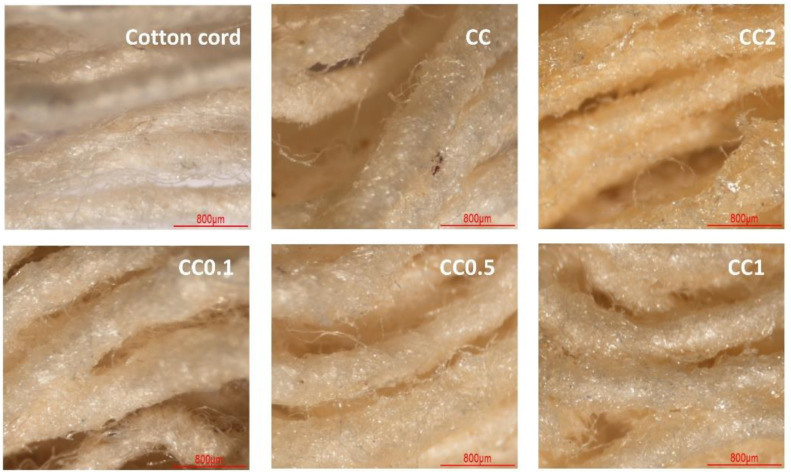
Physical properties of virgin cotton cord, CC, CC0.1, CC0.5, CC1, and CC2.

The physical appearance of the functionalized and virgin cotton rope was characterized by stereomicroscopy, as shown in Figure 4. The virgin cord appeared an ivory color. The CC, CC0.1, CC0.5, and CC1 systems displayed an ivory color with a slim layer containing polymer flakes on the rope surface. However, the CC2 systems demonstrated a pale-yellow color with a reflective polymer lamina on the cord surface according to the high quantity of PVOH, which was esterified at high temperatures and which increased the intensity of color.

**Figure 5:**
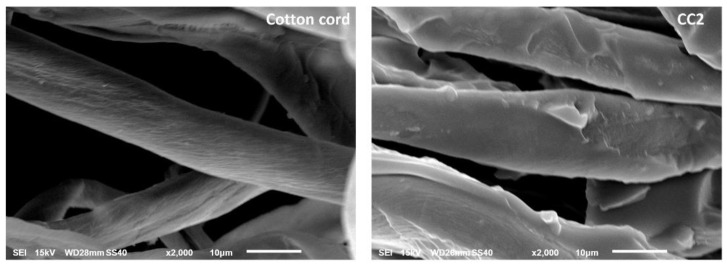
SEM images of virgin cotton cord and CC2.

The modified and virgin cotton ropes were also characterized by SEM to study the surface topography of samples, as illustrated in Figure 5. The virgin cord showed a smooth surface but the modified cord displayed a rough surface with a layer of the polymer coating.

#### 3.1.2. TGA Analysis

**Figure 6:**
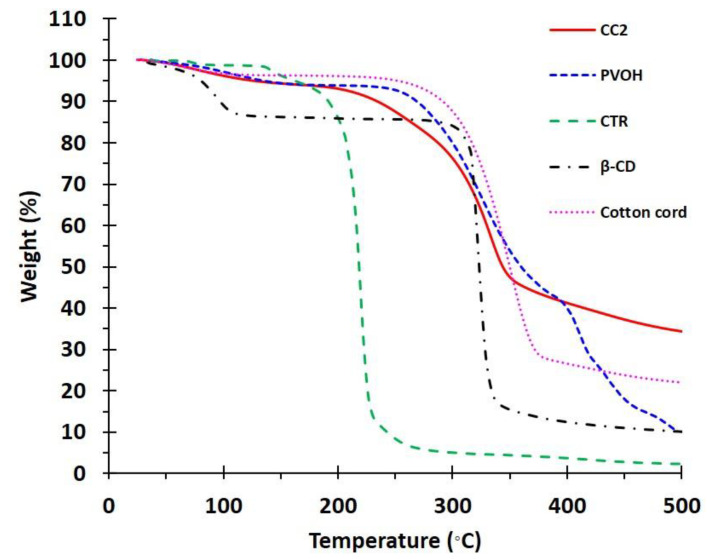
TGA thermograms of CTR, PVOH, cotton cord, CC2, and β−CD.

The thermal endurance of the functionalized cord was studied by TGA, as shown in Figure 6, for CTR, PVOH, cotton cord, CC2, and β−CD. The weight loss under 100 °C indicated the dehydration of the materials, which corresponded to a loss of 1.4%, 2.9%, 3.3%, 3.9%, and 10.7%, respectively. The thermal degradation began at 136.5 °C, 241 °C, 259 °C, 195.8 °C, and 296 °C, respectively. The residue over 500 °C was consistent with the final mass of 22.1% and 33.4% for cotton cord and CC2, respectively.

#### 3.1.3. ATR-FTIR Exploration

**Figure 7:**
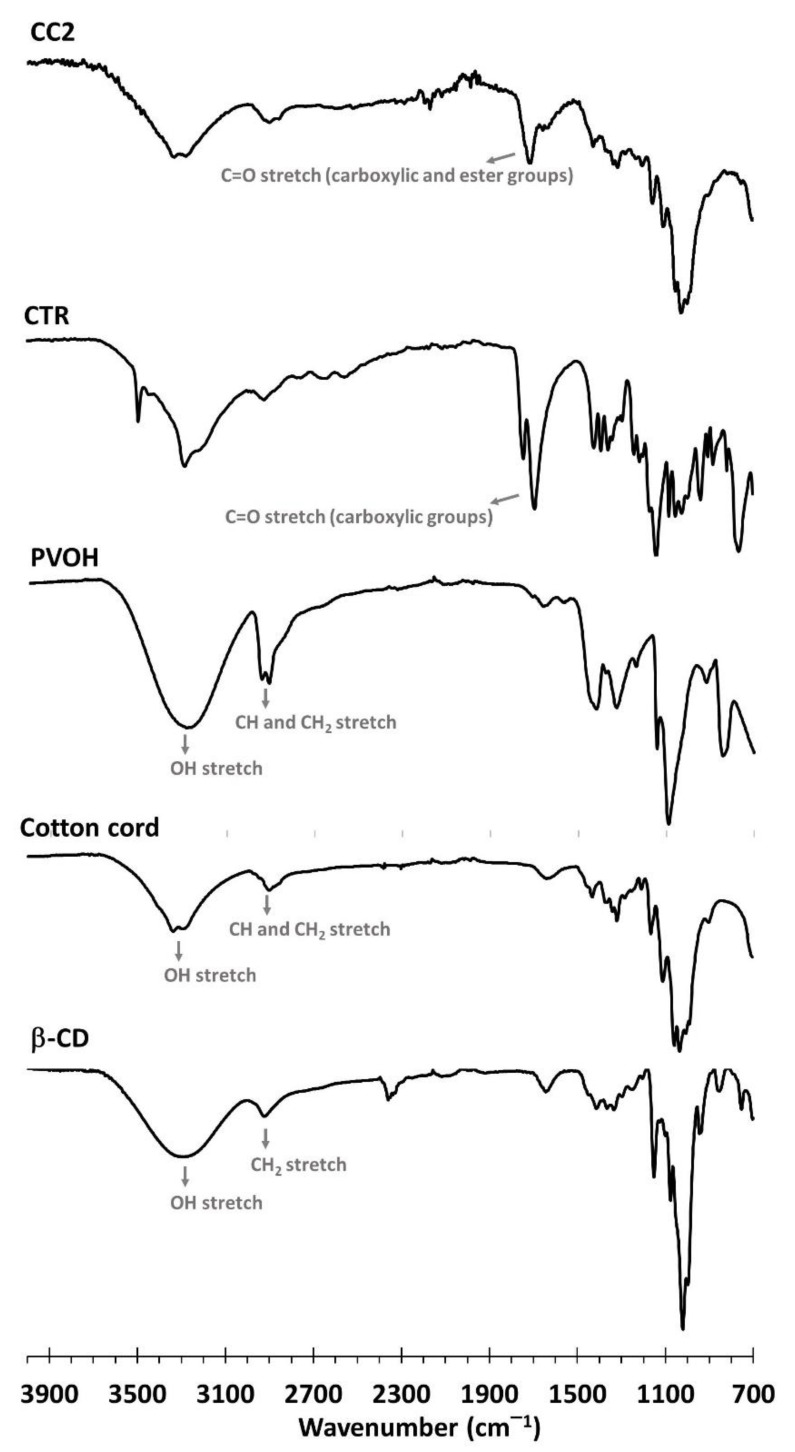
ATR-FTIR spectra of β−CD, cotton cord, PVOH, CTR, and CC2.

In Figure 7, The characterization of chemical groups on the functionalized cord was investigated by ATR-FTIR. The native β−CD spectra exhibited unique peaks at 3288 cm^−1^ (OH stretching) and 2917 cm^−1^ (CH_2_ stretching) [66]. The cotton cord spectra revealed specific peaks at 3298 cm^−1^(OH stretching) and 2896 cm^−1^ (CH_2_ stretching), as stated in the literature [67,68,69]. The PVOH spectra exhibited in particular unique peaks at 3267 cm^−1^(OH stretching), 2939 cm^−1^ (CH stretching), and 2907 cm^−1^ (CH_2_ stretching). The CTR spectra displayed specific revealed peaks at 1744 cm^−1^ and 1692 cm^−1^, attributed to the C=O stretching of carboxylic functions, as reported in previous works. The characteristic peak of CC2 at 1707 cm^−1^ corresponded to the C=O stretching of carboxylic and ester functions which were superposed to each other. Consequently, ATR-FTIR could prove the presence of ester bonds which confirmed the esterification between CTR and β−CD, PVOH, and/or cellulose, as informed in previous work [70].

#### 3.1.4. NMR Characterization

**Figure 8:**
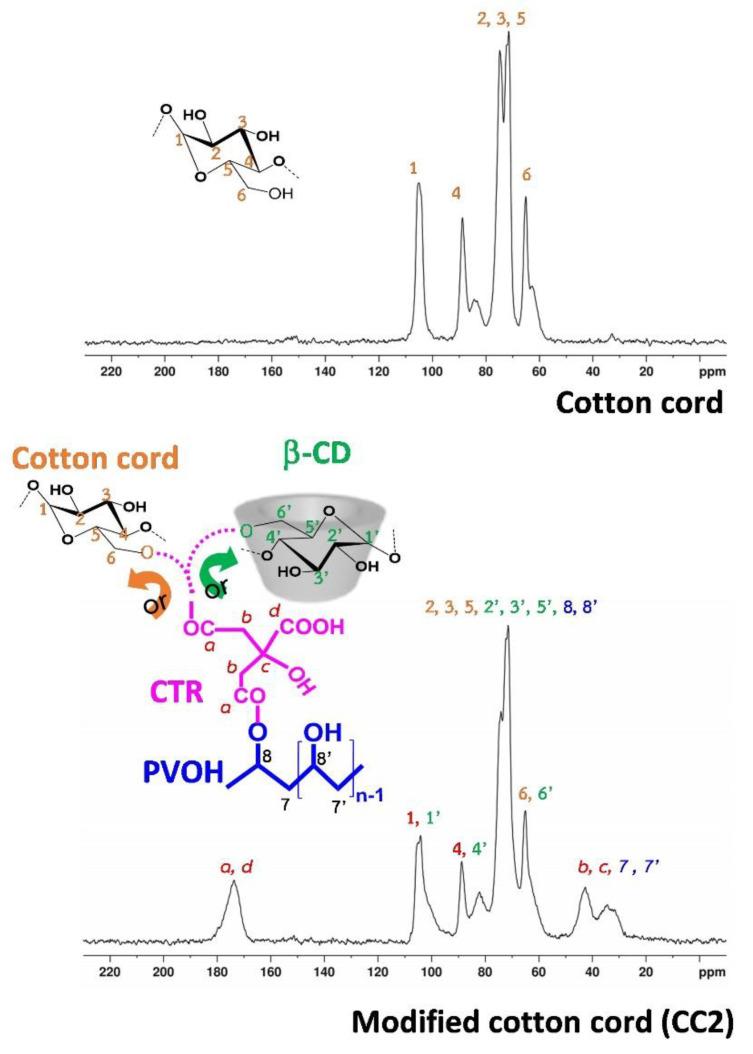
^13^C NMR spectra of the cotton cord and CC2.

The identification of the chemical structure of the modified cord and the virgin cotton cord was characterized by ^13^C NMR spectroscopy, as seen in Figure 8. The chemical shift of cotton cord was indicated as followed: at 65.0 ppm (for 6), 71.4 ppm (for 2 and 5), 74.7 ppm (for 3), 88.7 ppm (for 4), and 104.9 ppm (for 1). The ^13^C spectra of CC2 revealed specific peaks at 173.7 ppm (for a and d), 104.1 ppm (for 1 and 1′), 88.7 ppm (for 4 and 4′), 72.4 ppm (for 2, 3, 5, 8, 2′, 3′, 5′, and 8′), 64.9 ppm (for 6 and 6′), and 42.5 ppm (for b, c, 7, b’, c’, and 7′). Thus, the esterification reaction between CTR and β−CD, PVOH, and/or cellulose was approved by the slight change of chemical shift between unmodified and modified cotton cords, as reported in the literature [23].

### 3.2. Adsorption Study

#### 3.2.1. Preliminary Adsorption Study

The unique characteristic of both adsorbate and adsorbent, which are involved in the pH of pollutant solutions, affected the adsorption efficiency of β−CD (18 g/L of solubility in water at 25 °C), PQ (pH-independent and 620 g/L of solubility in water at 25 °C [21]), MB (a pKa value of 5.6 [71] and 43.6 g/L of solubility in water at 25 °C [72], and CV (a pKa_1_ value of 5.31 and a pKa_2_ of 8.64 and 4 g/L of solubility in water at 25 °C [44]).

**Figure 9:**
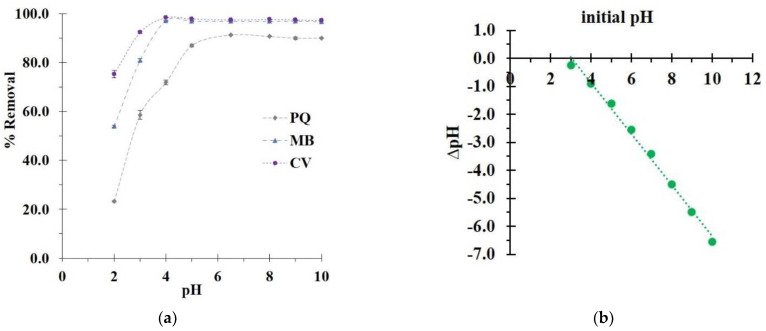
(**a**) Influence of pH on the pollutant removal (conditions: 5 g/L of adsorbent dosage, 25 mg/L of initial concentration, 360 min of contact time, and temperature at 303 K); (**b**) measurement of the point of zero charge (PZC) of modified cotton.

The optimization of the pH was firstly investigated, as shown in Figure 9a. The modified cord (CC2) revealed a low percentage of removal at pH 2 (23.2%, 54.0%, and 75.3% for PQ, MB, and CV removal, respectively), which might have occurred due to the host–guest interaction, network entrapment, and hydrogen bonding. Herein, the absence of electrostatic interaction caused a low adsorption, because the carboxylic groups of CTR could not be activated when the pH of the solution was smaller than the pKa of CTR (3.13, 4.76, and 6.40). After that, the percentage of removal increased with the pH until attaining a maximum at a pH of 6.5, 4, and 4 for PQ, MB, and CV adsorption, respectively, because the carboxylic groups of CTR could be dissociated to carboxylate functions when the pH of the solution was greater than some pKa values of CTR. This circumstance provided additionally an electrostatic interaction with pollutants, which could enhance the adsorption capacity. The pH of 6.5 was in agreement with the literature for CTR cross-linked with CD polymers for PQ removal [22]. The pH of 4 was in agreement with the literature for CTR cross-linked with CD polymers for MB removal [35].

As displayed in Figure 9b, the plot of ∆pH against the initial pH displayed a linear relationship (R^2^ = 0.9940) and a straight curve (y = −0.9113*x* + 2.7648), from which the point of zero charge (PZC) of the modified cord (CC2) was obtained from the intercept of this line curve at a pH of 2.8. At pH 2, the surface charge of the modified textile was positive (if pH < PZC) which provided a low adsorption efficiency, because of the repulsion between cationic pollutants and adsorbent. At a higher pH (more than 2.8), the surface charge became gradually negative (if pH > PZC), which could interact with cationic pollutants.

Moreover, the pKa values of MB and CV were also investigated for removal performance. When the pH of the solution became lower than their pKa values, these two species showed cationic charges by protonation, which could react with anionic charges from the adsorbent via electrostatic interaction to improve the adsorption efficiency. As a result, the optimal pH of the solution was 6.5, 4, and 4 for PQ, MB, and CV adsorption, respectively.

#### 3.2.2. Kinetics Study

**Figure 10:**
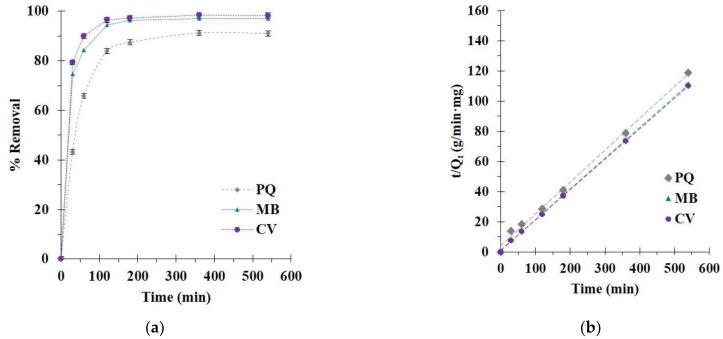
(**a**) Effect of contact time on pollutant adsorption; (**b**) pseudo-second-order kinetics of pollutant adsorption (conditions: 5 g/L of adsorbent dosage, 25 mg/L of pollutant initial concentration, optimal pH, and temperature at 303 K).

The kinetics of pollutant removal (PQ, MB, and CV) was studied at different contact times. The adsorption enhanced rapidly for the initial 180 min before attaining the plateau of adsorption at 360 min according to the unavailability of active sites, as noticed in Figure 10a. Consequently, an optimal contact time of 360 min was opted for the rest of the study.

The experimental data were subjected to the kinetic models to interpret the adsorption process dealing with the chemical reaction, adsorption order, and mass transfer. In Table 2, the correlation coefficients (R^2^) were larger for the pseudo-second-order model (R^2^ = 0.9968, 0.9997, and 0.9999) than for the pseudo-first-order model (R^2^ = 0.7766, 0.9552, and 0.9558) for PQ, MB, and CV removal, respectively. The correlation coefficient of the pseudo-second-order model was close to 1, which displayed as a straight line and confirmed the suitability of the model to the experimental data, as illustrated in Figure 10b. The adsorption efficiency was calculated by the pseudo-second-order model (Q_e,cal_ = 4.56, 4.86, and 4.91 mg/g for PQ, MB and CV removal, respectively). The Chi-square values for the pseudo-second-order model were inferior to those of the pseudo-first-order model for all systems, which also confirmed the reasonableness of the pseudo-second-order model with respect to the experimental data.

As observed in Table 3, the diffusion pathway was elucidated by the intraparticle diffusion configuration, which was separated into two parts: (i) the boundary layer diffusion relating to a fast removal rate constant for the first step (k_31_) and (ii) the intraparticle diffusion relating to a low removal rate constant for the second step (k_32_). Finally, the pollutant removal was a complex procedure because the curve of the two sections did not go through the origin.

#### 3.2.3. Isotherm Study

**Figure 11:**
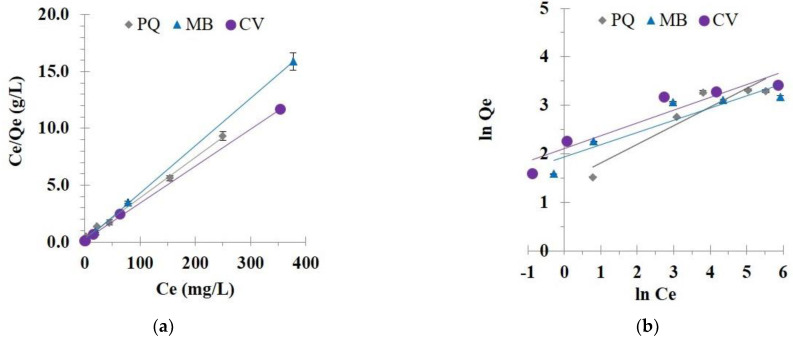
(**a**) Langmuir isotherm; (**b**) Freundlich isotherm of pollutant adsorption (conditions: 5 g/L of adsorbent dosage, 360 min of contact time, optimal pH, and temperature at 303 K).

The experimental data were consigned to the Langmuir (Figure 11a) and Freundlich (Figure 11b) isotherm models at 30 °C with different initial concentrations of pollutants to evaluate these models’ reasonableness.

The correlation coefficient (R^2^) was higher for the Langmuir isotherm model (R^2^ = 0.9969, 0.9999, and 0.9996) than for the Freundlich isotherm model (R^2^ = 0.8816, 0.8413, and 0.8878) for PQ, MB, and CV adsorption, respectively, as seen in Table 3. The linearity of the Langmuir isotherm model (R^2^ near 1) was achieved for the pollutant removal, which endorsed the adequacy of the model with the experimental data and explained the monolayer adsorption for pollutants on the modified cord surface. The Chi-square values for the Langmuir model were smaller than those of the Freundlich isotherm model for all systems, which also confirmed the suitability of the Langmuir isotherm with respect to the experimental data. The separation factor (R_L_) was between 0 and 1 for the PQ (0.292, 0.171, 0.064, 0.040, and 0.033 for the initial concentration of 25, 50, 150, 250, and 300 mg/L), MB (0.119, 0.063, 0.022, 0.013, and 0.011 for the initial concentration of 25, 50, 150, 200, and 500 mg/L), and CV removal (0.150, 0.081, 0.029, 0.017, and 0.014 for the initial concentration of 25, 50, 150, 200, and 500 mg/L). These values were reduced with the enhancement of the initial concentrations, which displayed a vigorous affinity between the modified cord and pollutants.

**Figure 12:**
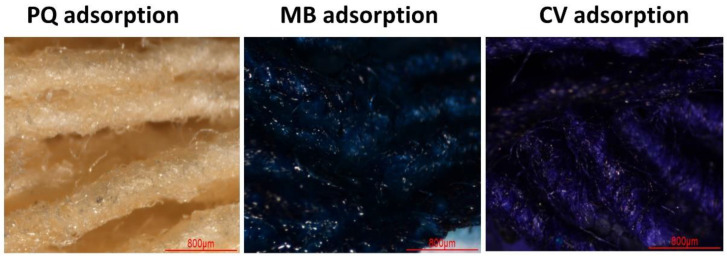
Illustration of the modified cotton (CC2) after PQ, MB, and CV adsorption.

In Table 3, the maximum adsorption capacity from the Langmuir model was equal to 28.3, 23.9, and 30.6 mg/g for PQ, MB, and CV adsorption, respectively. As seen in Table 4, the removal of PQ from CC2 was quite good, compared with the other adsorbents. However, the removal of MB and CV was low, compared with various materials. Although the removal performance was mediocre, it could be recycled many times using a suitable solvent. To valorize this modified cotton, it could be applied in other adsorption processes such as a semipilot scale, a continue system, or others. As illustrated in Figure 12, no different change was observed after PQ adsorption. The modified textile (CC2) showed blue color and violet color after MB and CV adsorption, respectively.

#### 3.2.4. Stability Study

**Figure 13:**
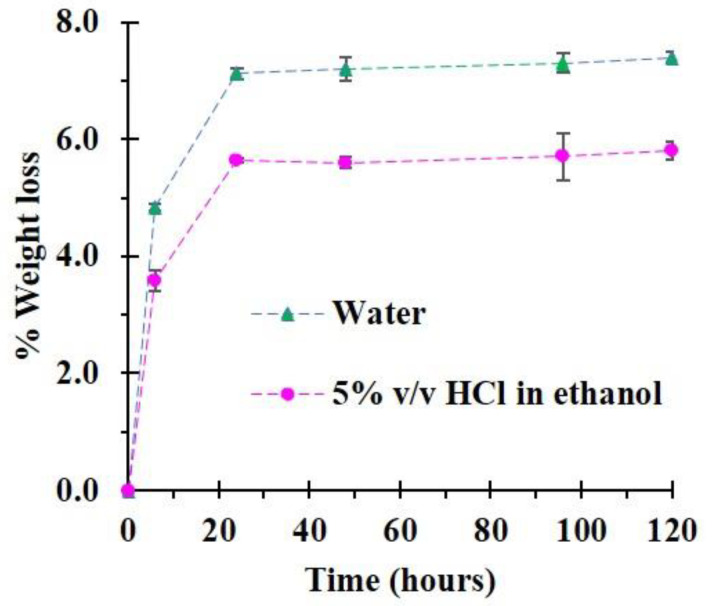
Stability of the modified cotton (CC2) in water and 5% *v*/*v* of HCl in ethanol.

The stability study of the modified cotton rope was operated in water and 5% *v*/*v* of HCI in ethanol to assess the endurance of the polymer coating during the adsorption and desorption processes, as shown in Figure 13. The weight loss of CC2 was observed after 6 h of contact time (4.8% and 3.6% for water and 5% *v*/*v* of HCI in ethanol, respectively). The degradation of CC2 was continued after 24 h of contact time, which was equal to 7.1 and 5.6%, successively. The coating was slightly degraded because the anionic cyclodextrin polymer was water-soluble and decayed comfortably in water via the breaking of ester bonds presented on the polymer structure through hydrolysis. This result immediately provided a drop in recyclability performance, which was found in previous works [20,73]. Nevertheless, the weight loss of 5% *v*/*v* of HCI in ethanol was smaller than in water.

#### 3.2.5. Reusability Study

**Figure 14:**
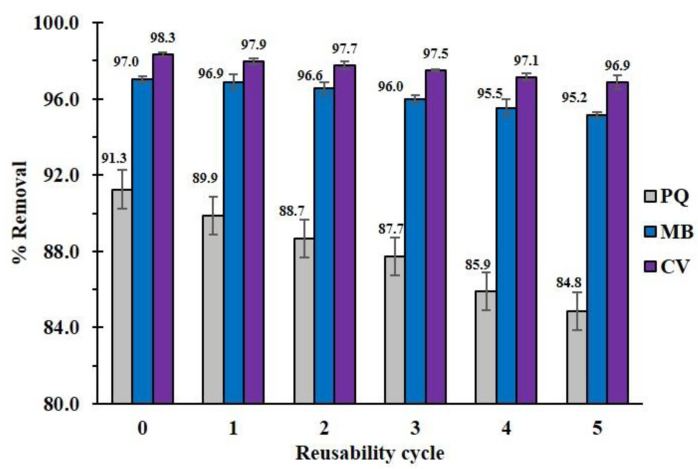
Reusability of the modified cotton (CC2) for pollutant removal.

The recyclability of the modified cord was investigated to estimate the cost-effectiveness of the adsorption method. In Figure 14, the reusability efficiency dropped after five uses (PQ (from 91.3% to 84.8%), MB (from 97.0% to 95.2%), and CV (from 98.3% to 96.9%)). This diminution of pollutant removal might be due to the decomposition of the polymer coating on the cotton surface after contact with solvents, as explained in the stability study.

## 4. Conclusions

The coating of anionic cyclodextrin polymer was achieved by in situ polymerization between β-cyclodextrin and citric acid in the presence of poly (vinyl alcohol) at 140 °C and 90 min. The modified cotton rope (CC2) revealed 79.2% coating performance, 1.12 mmol/g COOH groups, 91.3% PQ removal, 97.0% MB removal, and 98.3% CV removal for 25 mg/L of initial concentration. Various characterization methods were employed to confirm the physicochemical properties of modified rope. The adsorption of cationic pollutants on the modified cord was presented as four possibilities: host–guest interaction, electrostatic interaction, polymer network entrapment, and hydrogen bonding. Thus, the presence of poly (vinyl alcohol) provided supplementary adsorption sites to enhance the pollutant removal efficiency. The pseudo-second-order model and the Langmuir isotherm were appropriate for the kinetics and isotherm study, respectively. The adsorbent was stable after 24 h of contact time in water and 5% *v*/*v* of HCI in ethanol. After five regeneration of the modified rope, the adsorption rate was 84.3% (PQ), 95.2% (MB), and 96.9% (CV). This environmentally friendly material could be applied as an effective adsorbent for cationic contaminants from an aqueous solution and the coating process could be utilized on various supports for different kinds of applications.

## Figures and Tables

**Table 1:** Different formulations for cord coating.

Name	Composition (% *w*/*v*)		
	β−CD	CTR	PVOH
C2.5C	10	2.5	-
C2.5C2	10	2.5	2
C5C	10	5	-
C5C2	10	5	2
CC	10	10	-
CC0.1	10	10	0.1
CC0.5	10	10	0.5
CC1	10	10	1
CC2	10	10	2

**Table 2:** Pseudo-second-order and pseudo-first-order kinetics parameters (conditions: 5 g/L of adsorbent dosage, 25 mg/L of initial concentration, optimal pH, and temperature at 303 K).

	Q_e (exp)_	Pseudo-First-Order				Pseudo-Second-Order						Adsorption Mechanism	
		R^2^	Q_e (cal)_	χ^2^	k_1_	R^2^	Q_e (cal)_	χ^2^	k_2_	h	t_1/2_	k_31_	k_32_
PQ	4.56	0.7766	1.92	3.6311	0.0124	0.9968	4.73	0.0059	0.0118	0.3	18.0	0.3656	0.0191
MB	4.86	0.9552	1.56	6.9771	0.0205	0.9997	4.92	0.0006	0.0372	0.9	5.5	0.1793	0.0037
CV	4.91	0.9588	1.20	11.5611	0.0184	0.9999	4.95	0.0003	0.0509	1.2	4.0	0.1524	0.0053

**Table 3:** Langmuir and Freundlich isotherm parameters (conditions: 5 g/L of adsorbent dosage, 360 min of contact time, optimal pH, and temperature at 303 K).

	Q_e (exp)_	Langmuir Isotherm			Freundlich Isotherm					
		R^2^	Q_m (cal)_	K_L_	χ^2^	R^2^	Q_m (cal)_	K_f_	1/n	χ^2^
PQ	26.9	0.9969	28.3	0.10	0.077	0.8816	14.8	4.2	0.39	9.871
MB	23.7	0.9999	23.9	0.30	0.001	0.8413	15.5	7.0	0.25	4.423
CV	30.3	0.9996	30.6	0.23	0.003	0.8878	20.3	8.2	0.26	4.886

**Table 4:** Langmuir isotherm for pollutant removal by various adsorbents.

Adsorbent	Adsorption Dosage	Paraquat Concentration (mg/L)	Maximum Adsorption Capacity
**PQ removal**			
CTR-CD-PVOH coated on cotton rope (this work)	0.05 g in 0.01 L	25–300 mg/L	26.9 mg/g
CTR-CD-PVOH nanosponges [23]	0.02 g in 0.01 L	25–300 mg/L	112.2 mg/g
CTR-CD coated on polyester textile [20]	0.02 g in 0.01 L	10–200 mg/L	21.9 mg/g
Bentonite [14]	0.04 g in 0.025 L	4–24 mg/L	11.75 mg/g
Activated carbon [13]	0.01 g in 0.01 L	1.5–45 mg/L	20 mg/g
**MB removal**			
CTR-CD-PVOH coated on cotton rope (this work)	0.05 g in 0.01 L	25–500 mg/L	23.7 mg/g
CTR-CD polymer [35]	0.2 g in 0.2 L	4–1000 mg/L	248 mg/g
CTR-CD modified filter paper [33]	0.1 g in 0.1 L	50–500 mg/L	124.6 mg/g
CTR-CD modified wood flour [34]	0.25 g in 0.05 L	100–1000 mg/L	86.2 mg/g
CTR-CD-silica hybrid adsorbent [30]	0.01 g in 0.01 L	25–1250 mg/L	181.1 mg/g
**CV removal**			
CTR-CD-PVOH coated on cotton rope (this work)	0.05 g in 0.01 L	25–500 mg/L	30.3 mg/g
EDTA/graphene oxide functionalized corncob [47]	0.01 g in 0.025 L	20–140 mg/L	203.9 mg/g
Modified rice husk [41]	0.02 g in 0.01 L	20–100 mg/L	97.7 mg/g
CD functionalized magnetic adsorbent [46]	0.005 g in 0.002 L	300–1500 mg/L	454.5 mg/g
EDTA/CD polymers [48]	0.01 g in 0.005 L	10–500 mg/L	114.2 mg/g

## Data Availability

The study did not report any data.
